# Identification of volatile compounds and evaluation of certain phytochemical properties of Turkish propolis

**DOI:** 10.1002/fsn3.4399

**Published:** 2024-08-12

**Authors:** Berfin Yavaş, Ayhan Baştürk

**Affiliations:** ^1^ Department of Food Engineering, Faculty of Engineering Van Yüzüncü Yıl University Van Turkey

**Keywords:** antioxidant activity, clustering, PCA, propolis, total phenolic content, volatile component

## Abstract

The purpose of this study was to assess volatile component profiles and the antioxidant activity of propolis samples from eight different locations in Türkiye. α‐Pinene, β‐pinene, 3‐carene, limonene, 2‐acetylfuran, benzaldehyde, acetic acid, benzoic acid, longifolene, isopentyl acetate, m‐cymene, styrene, and δ‐cadinene were the most common volatile components found in the most of propolis samples. Principal component analysis (PCA) and hierarchical cluster analysis were performed on the gas chromatography–mass spectrometry (GC–MS) data to identify trends and clusters in the propolis samples. As a result of the PCA, the common components in all propolis were m‐cymene, decanal, α‐pinene, limonene, pinocarvone, benzaldehyde, and butanoic acid. The samples had 2,2‐diphenyl‐1‐picrylhydrazyl radical (DPPH) inhibition activity ranging from 19.2% to 92.5% and 2,2′‐azinobis(3‐ethylbenzothiazoline‐6‐sulfonic acid) (ABTS) values ranging from 480 to 1370 μM trolox/g extract. The total phenolic content (TPC) ranged from 10,900 to 34,033 mg GAE/100 g. Compared with butylated hydroxytoluene (BHT) (control), all but one of the propolis samples exhibited higher DPPH activity. In addition, ABTS levels of propolis extracts were higher than those of BHT. These results unequivocally show that Turkish propolis has remarkable antioxidant qualities, which makes it a viable option for addition to food and medicine products.

## Graphical abstract

The total phenolic content (TPC) of the Turkish propolis under investigation was generally high. Compared to butylated hydroxytoluene (BHT), six of the eight samples had greater antioxidant activity. Propolis from various parts of Turkey contains 61 different components. Principal component and hierarchical cluster analyses were performed on the propolis samples.

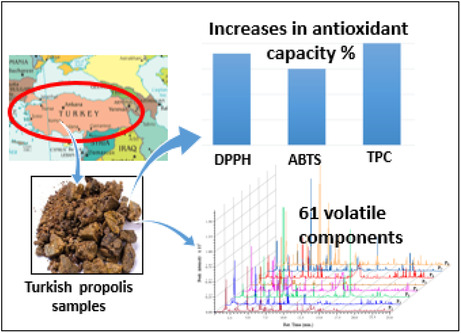

## 1 INTRODUCTION

The natural resinous substance known as propolis is collected from honeybees (*Apis mellifera* L.) using various plant sources. Published research indicates that propolis has a varied and intricate chemical structure. Propolis is composed of over 300 bioactive substances, including volatile organic compounds, phenolic acids, vitamins, amino acids, terpenes, sterols, and their esters (Graikou et al., 2016; Mehmetoğlu et al., 2017). The chemical composition of propolis is directly influenced by its geographical location, season, altitude, local flora type, and climatic conditions (Bankova, 2005; Cheng et al., 2013b; Soltani et al., 2017). Therefore, propolis may have different antioxidant activities, total phenolic content (TPC), phenolic compounds, and volatile component profiles.

**FIGURE 1:**
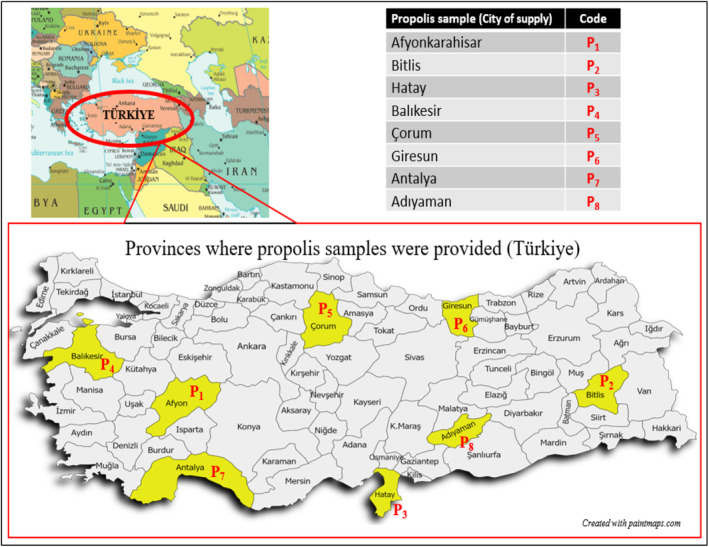
Map of Türkiye indicating the provinces where propolis samples were collected and sample codes.

Propolis, collected by bees from plants, contains antioxidant compounds, and interest in this product is increasing day by day. Because of the recent COVID‐19 pandemic, customers believe that it is safe to utilize nontoxic propolis as an alternative preventive agent because of its antioxidant, antibacterial, and immune‐boosting properties. Consequently, propolis has become increasingly popular in the commercial world. It can be used in food additives, cosmetics, and over‐the‐counter products. Over the past 50 years, propolis has been shown to possess a wide range of biological properties, including antibacterial, antifungal, antiviral, cytotoxic, antioxidant, and anti‐inflammatory properties (Banskota et al., 2001; Sforcin & Bankova, 2011). Despite the fact that propolis contains very low levels of volatile chemicals, its pleasant scent and biological activity make it useful for characterizing the substance (Bankova et al., 2000). According to several studies (Haile et al., 2012; Kaškonienė et al., 2014; Melliou et al., 2007; Pino et al., 2006), the volatile compounds present in propolis are highly variable and depend on the specificity of the local flora, geographical and climatic characteristics in the region where the propolis was collected (Bankova, 2009). Therefore, research on the chemical structure of propolis collected from different locations should be conducted. Türkiye, or Anatolia, is a vast geographical area with a wide variety of subtropical floras. Anatolia is home to almost 11,000 native plant species (Guzelmeric et al., 2018). The diversity of species found in different parts of the world makes it impossible to define propolis in terms of chemical composition. The literature contains a few investigations that have been conducted on Anatolian propolis (Altuntaş et al., 2023; Arserim‐Ucar et al., 2020; Arslan et al., 2021; Can et al., 2024; Ecem Bayram, 2015; Erdogan et al., 2011; Hames‐Kocabas et al., 2013; Kolaylı et al., 2023; Sahinler & Kaftanoglu, 2005; Sarıkahya et al., 2021; Uzel et al., 2005). The biological properties (antioxidant, antibacterial, antifungal, etc.) and chemical constituents (phenolic and volatile chemicals) of propolis collected from various parts of Türkiye were studied by these scientists. Sarıkahya et al. (2021) found α‐pinene, β‐pinene, and limonene as main volatile compounds in most of the propolis samples collected from 39 different regions in Türkiye. In a study on propolis from Bingöl (Türkiye), it was reported that the presence of bioactive components in the lipophilic and essential oil isolates of Bingöl propolis contributes to its biological activity and has the potential to be used as a natural preservative in food systems (Arserim‐Ucar et al., 2020). Altuntaş et al. (2023) characterized the biochemical composition and biological activity of 24 propolis samples from different regions of Turkey according to their geographical origin. Can et al. (2024) investigated various properties (total phenolic content, total flavonoid values, condensed tannin content, and phenolic profiles of ethanolic extracts) of crude propolis from the Marmara Region of Türkiye. They reported that propolis from the Marmara Region is a rich source of bioactive compounds, especially flavonoids, with antioxidant properties. Bayram et al. (2018) analyzed 64 propolis samples from the southeastern region of Türkiye. They emphasized that propolis and other bee products should not be allowed to be sold in the market without safety and toxicity information and full chemical composition data. The chemical composition of propolis varies depending on factors, such as the type of plant the bee reaches, geographical conditions, time and method of propolis collection, and the extraction method applied. Propolis samples were collected from the provinces of Bitlis, Hatay, Balıkesir, Çorum, Giresun, Antalya, and Adıyaman in Türkiye. Figure 1 shows the provinces and codes in which the propolis was collected. This study includes data on the volatile component profiles and antioxidant activity. In addition, the total phenolic contents of the propolis were compared. By comparing the data obtained from propolis from eight different regions, we investigated how the chemical composition and biological activities vary according to the region.

## 2 MATERIALS AND METHODS

### 2.1 Materials

Propolis was collected between May and October 2019 from eight regions of Türkiye: Afyonkarahisar, Bitlis, Hatay, Balıkesir, Çorum, Giresun, Antalya, and Adıyaman. A single provider provided the propolis samples for every city. Figure 1 shows the Turkish provinces where propolis was gathered, together with the sample codes. Following collection, the propolis was stored at −18°C in sealed bags until analysis. Three duplicate analyses were performed. Butylated hydroxytoluene (BHT), Folin–Ciocalteu reagent, 2,2‐diphenyl‐1‐picrylhydrazyl (DPPH), 2,2‐azinobis(3‐ethylbenzothiazoline‐6‐sulfonic acid) diammonium salt (ABTS), Trolox (≥95%), and gallic acid (GA) (≥98%) were obtained from Sigma‐Aldrich (St. Louis, MO, USA). All the other chemicals used in the analysis were of analytical grade.

### 2.2 Methods

#### 2.2.1 Purification of propolis from oils

The frozen samples were finely pulverized using a mortar and pestle, prior to the extraction procedure. They were then subjected to extraction using n‐hexane in a Soxhlet apparatus. The oil and waxy components of propolis were removed by extraction. Defatted propolis samples were dried in the shade and prepared for methanol extraction.

#### 2.2.2 Extraction of propolis

Ten grams of nonfat powdered propolis was homogenized in 50 mL methanol with a homogenizer (Heidolph, Silent Crusher M, Schwabach, Germany) at 10,000 rpm (revolutions per minute) for 15 seconds. After adding 5 μL hydrochloric acid (HCl), the mixture was shaken for 3 h at room temperature at 200 rpm on a circular shaker (Heidolph, Unimax 1010, Kelheim, Germany). It was then filtered through Whatman filter paper (No. 4), and the filtrate was transferred to Falcon tubes and centrifuged at 8000 × *g* for 20 min (4°C). After filtering again using Whatman filter paper, the supernatant was placed in a separate container. After centrifugation, 10 mL methanol was added to the residue remaining at the bottom of the Falcon tube, and the same procedure was repeated. The resulting supernatants were combined and the methanol content was removed using a rotary evaporator (Heidolph, Germany) at 50°C. Powdered extracts were weighed and diluted in methanol to a final volume of 25 mL.

#### 2.2.3 Determination of total phenolic content

The total phenolic content was determined using the Folin–Ciocalteu technique (Singleton & Rossi, 1965). Test tubes were filled with 0.4 mL of the extract and diluted using Folin's solution (2 mL). The liquid was gently shaken before 1.6 mL of a 7.5% sodium carbonate solution was added. After the tubes were sealed, the mixtures were thoroughly shaken to achieve homogeneity. The samples were then allowed to sit at room temperature in the dark for an hour. The absorbance was measured at 760 nm against a blank (solution with no extract) using a spectrophotometer. All extracts were applied to gallic acid (GA) as a standard curve (*y* = 0.003*x* + 0.024, *R* ^2^ = 0.998) and the absorbance was measured at 760 nm. The results were expressed as milligrams of gallic acid equivalents per kilogram (mg GAE/kg).

#### 2.2.4 Determination of DPPH activity

With a few adjustments, the method of Huang et al. (2005) was used to conduct the DPPH (2,2‐diphenyl‐1‐picrylhydrazyl) experiment. A mixture of 0.1 mL of extract and 3.9 mL of DPPH solution (0.025 g/L methanol) was prepared. The mixture was kept at room temperature in the absence of light for 120 min. The absorbance of each sample was measured at 515 nm and compared to that of the blank sample. The DPPH radical inhibition rate was calculated using the DPPH radical equation:
(1)

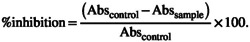




#### 2.2.5 ABTS radical cation decolorization assay

The 2,2′‐azinobis(3‐ethylbenzthiazoline‐6‐sulfonic acid) (ABTS) free‐radical‐scavenging activity of each sample was assessed by modifying the procedure outlined by Re et al. (1999). To create a stock ABTS+ radical solution, a 7 mM ABTS^+^ solution containing 2.45 mM potassium persulfate (K_2_S_2_O_8_) was prepared and allowed to stand at room temperature in the dark for 12–16 h. The stock radical solution was diluted with a mixture of water and ethanol (1:1, v/v) to obtain ABTS^+^ solution. The absorbance of the ABTS^+^ solution was adjusted to 0.70 ± 0.02 at 734 nm. The extract (20 μL) was reacted with 1980 μL ABTS^+^ solution. After 6 min, absorbance was measured at 734 nm. A Trolox standard curve was used to calculate the results (μM Trolox/g extract) (*y* = 0.338*x* + 2.7409, *R* ^2^ = 0.995). All experiments were performed in triplicate.

#### 2.2.6 Volatile component analysis

Propolis samples from several provinces were analyzed for volatile components using the gas chromatography–mass spectrometry (GC–MS) solid‐phase microextraction (SPME) method. With a few minor adjustments, the procedures suggested by Krist et al. (2006) were used to conduct the analyses. 5‐methyl 2‐hexanone (Merck‐Schuchardt OHG) was used as an internal standard. Three grams of raw propolis powder was weighed, and 10 mL of pure water that had been boiled and cooled was added. The mixture was then homogenized at 13,000 revolutions per minute using a homogenizer (Heidolph Silent Crusher M, Schwabach, Germany). Twenty microliters of the refrigerated standard solution was added, and a magnetic fish was placed in the vial. The vial was placed in a heating block set to 40°C at a rotation speed of 250 rpm and conditioned for 5 min. At the end of the experiment, the fiber (50/30 μm thick, divinylbenzene/carboxen/polydimethylsiloxane (DVB/CAR/PDMS) as adsorbent, Supelco, Bellefonte, PA, USA) was immersed into the vial and the volatile components in the headspace were adsorbed for 30 min. The fiber was then removed from the vial and kept in the injection port of the GC/MS device for 5 min to allow the adsorbed volatile components to pass through the device column. RTX‐5MS (30 m long, 0.25 mm inner diameter, and 0.25‐μm film thickness) capillary column was used for GC–MS. The operating conditions were set as follows: column oven temperature, 40°C; injection mode, split; injection temperature, 250°C; and total flow rate: 20.7 mL/min.; and column flow rate: 1.61 mL/min., oven progress: 40°C (2 min), 4°C/min from 40 to 230°C (3 min); MS conditions: ion source temperature, 200°C; interface temperature, 250°C; solvent cut time, 1 min; and electron ionization (EI), 70 eV. The compounds were identified by cross‐referencing the published literature, mass spectrum libraries from NIST 17 (National Institute of Standards and Technology) (Standard Reference Data Program Gaithersburg) and Wiley 9 (Wiley, New York, NY, USA), and retention indices (RI), which were calculated in relation to the retention times of n‐alkanes (C9–C25). Proportional and quantitative calculations were performed for each volatile component. The identified components’ quantities (μg/kg) were computed using the mass spectra of the internal standard and the components.

#### 2.2.7 Statistical analysis

SPSS (version 20.0; IBM Corp., SPSS Inc., NY, USA) was used to assess the study's findings. To ascertain whether there was a statistically significant difference between the group averages, one‐way analysis of variance (ANOVA) was employed. Duncan's multiple comparison test was used to determine the significance level of differences. STATISTICA (StatSoft, Inc. 2007, version 8.0) was used to create PCA, clustering tests, and graphs.

## 3 RESULTS AND DISCUSSION

### 3.1 Total phenolic content

**FIGURE 2:**
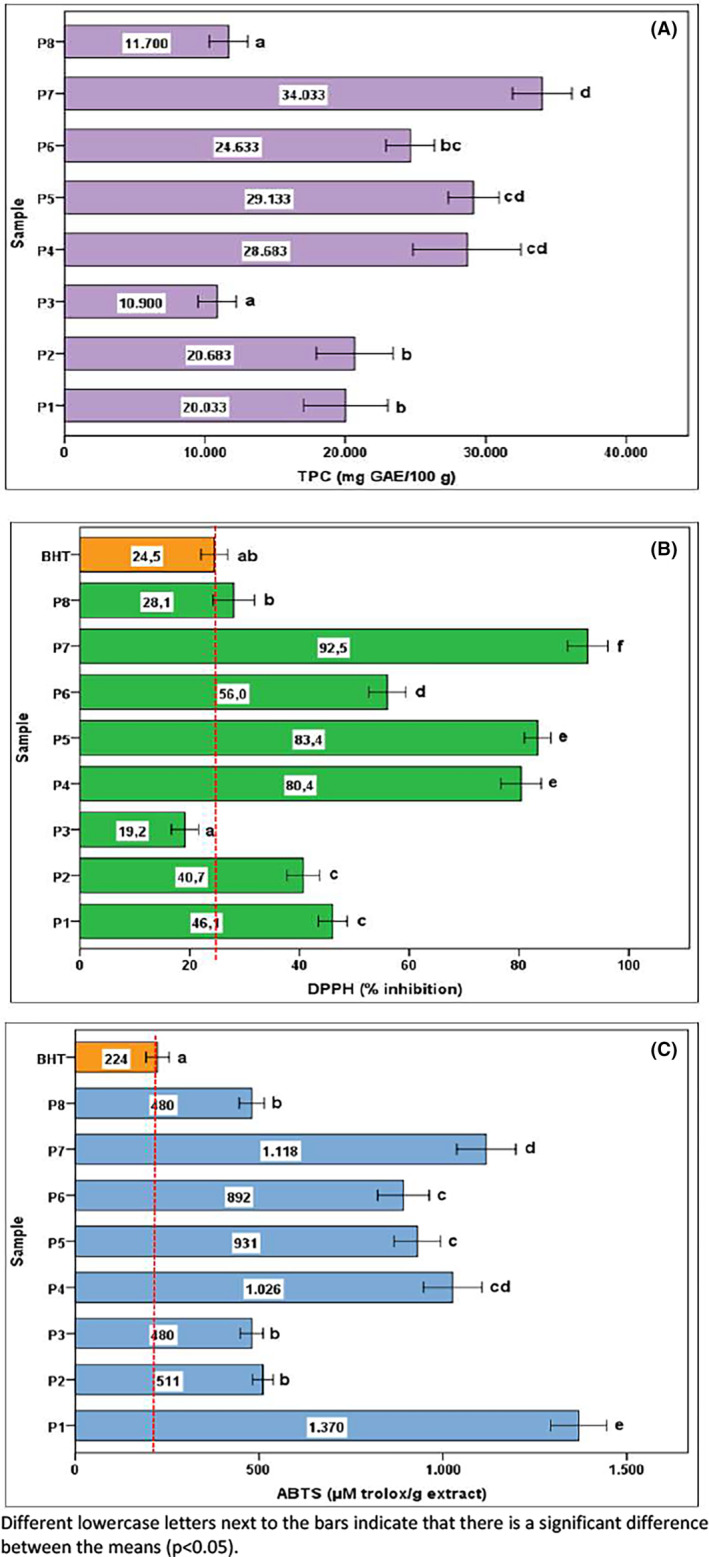
Total phenolic content (TPC), DPPH, and ABTS analysis results of propolis samples. Different lower case letters next to the bars indicate that there is significant difference between the means (*p* < .05).

Figure 2A displays the total phenolic content (TPC), which is thought to be one of the factors influencing antioxidant activity. The TPCs of the samples ranged from 10,900 to 34,033 mg GAE/100 g (*p* < .05). P_3_ and P_8_, P_1_ and P_2_, and P_4_ and P_5_ were close to each other, and there were no significant differences between them. The highest TPC was observed at P_7_ (34,033 mg GAE/100 g). P_3_ and P_8_ exhibited the lowest TPC. Degirmencioglu et al. (2019) determined TPCs in 19 distinct crude propolis samples taken from five different locations in Türkiye (Aegean, Black Sea, Central Anatolia, Marmara, and Mediterranean locations) within the range of 1124–17,298 mg GAE/100 g. Can et al. (2024) determined TPCs in the range of 5290–20,350 mgGAE/100 g in propolis collected from 11 different locations in the Marmara Region of Türkiye. TPC in propolis samples from various parts of Türkiye ranged from 2880 to 8040 mg GAE/100 g, according to a previous study by Keskin et al. (2020). TPC was determined as 418–12,575 mg GAE/100 g in propolis obtained from the Tunceli Province of Türkiye (Akman et al., 2023). Altuntaş et al. (2023) determined TPCs to be between 1673 and 12,583 mg GAE/100 g in propolis collected from 24 different locations in Anatolia. Our findings were generally higher than those reported in the literature results. TPCs in Venezuelan, Argentine, and Brazilian propolis have been reported to be 1910–10,700, 17,600–18,200, and 15,800 mg GAE/100 g, respectively (Mohtar et al., 2020). In a total of 13 propolis samples from different geographical regions, six from northeastern Europe, two from the tropical region of South America, and five from South‐Western Europe, TPCs varied between 6549 and 22,840 mg GAE/100 g (Osés et al., 2020). The chemical composition of propolis is influenced by factors, such as geographical location, season, altitude, local flora, climatic conditions, collection method, and extraction technique. Therefore, propolis might have different TPC values.

### 3.2 Antioxidant activity

The DPPH and ABTS assays were used to assess the antioxidant activity of the methanolic extracts of propolis. The BHT was used as a reference. Figure 2B shows the DPPH% inhibition values for propolis collected from eight distinct areas. The propolis samples showed inhibition effects in the range of 19.292.5%. There was no significant difference between P1 and P2 and between P4 and P5, whereas there was a significant difference between the other samples' mean % inhibition values. All samples, except P3, showed higher DPPH% inhibition value than BHT. In particular, propolis coded P7, P4, and P5 had approximately three and a half times higher inhibition effect than BHT. The propolis extract from the Finike area of Antalya, Türkiye had a DPPH% inhibition value of 65.67% (17.15 mM trolox. eq/g) according to Ceylan and Basturk (2022). Sarıkahya et al. (2021) reported DPPH% inhibition values of propolis from different geographical regions of Türkiye in the range of 55.98–86.17%. Similar findings have been reported in other studies that have evaluated propolis from different geographical regions (Kalogeropoulos et al., 2009; Mohdaly et al., 2015; Nina et al., 2015). Our conclusions are broadly supported by the data from these investigations. In addition, Kolaylı et al. (2023) reported that propolis from the Aegean region showed the lowest DPPH activity (antioxidant activity (SC50), 7.50 μg/mL) among propolis from seven different geographical regions of Anatolia. The chemical compositions of the propolis samples differed because they were collected from various areas around Türkiye. Depending on the botanical and geographic origin of propolis, these components may change. Given that propolis sourced from distinct phytogeographic locations within our nation displays significantly distinct chemical profiles, it is reasonable to anticipate the DPPH differences (*p* < .05) observed among the samples.

As shown in Figure 2C, ABTS radical‐scavenging activities detected in propolis samples were determined to be between 480 and 1370 μmol trolox/g (120.1–342.9 mg trolox/g) (*p* < .05). The ABTS value of BHT was lower than that of the propolis. There were no significant differences among P2, P3, and P8. P1 showed the highest ABTS radical‐scavenging activity, which was approximately six times higher than that of BHT. Degirmencioglu et al. (2019) determined ABTS values as 24.94–309.81 mg trolox/g in 19 different crude propolis samples collected from five different regions of Türkiye (Aegean, Black Sea, Central Anatolia, Marmara, and Mediterranean Regions). The ABTS values of Spanish propolis were found by Oses et al. (2016) to be between 1184.66 and 1400.86 μmol trolox/g. These values were 52–409, 429–431.5, and 420.9 μmol trolox/g in Venezuelan, Argentinian, and Brazilian propolis, respectively (Mohtar et al., 2020). Our results, together with a literature review, indicate that propolis exhibits a variety of antioxidant properties. Various factors, such as the geographical location and flora from which the propolis was collected, the time of collection, the collection methods employed, and the extraction techniques used, may have contributed to the observed differences.

### 3.3 Correlation between DPPH, ABTS, and TPC

The correlations between DPPH, ABTS, and TPC of the propolis samples are presented in Table 1. As shown in the graph, there is a strong positive correlation between these three parameters. Strong positive correlations were observed between TPC and DPPH at 0.01 level (0.935), TPC and ABTS at 0.05 level (0.615), and DPPH and ABTS at 0.05 level (0.698). In other words, an increase in TPC increased DPPH activity more than ABTS activity.

**TABLE 1:** Correlation between DPPH, ABTS, and TPC.

Pearson correlation	DPPH	ABTS	TPC
DPPH	1	.698**	.935**
ABTS	.698**	1	.615*
TPC	.935**	.615*	1

** Correlation is significant at the 0.01 level (2‐tailed).

* Correlation is significant at the 0.05 level (2‐tailed).

### 3.4 Volatile components of propolis

**FIGURE 3:**
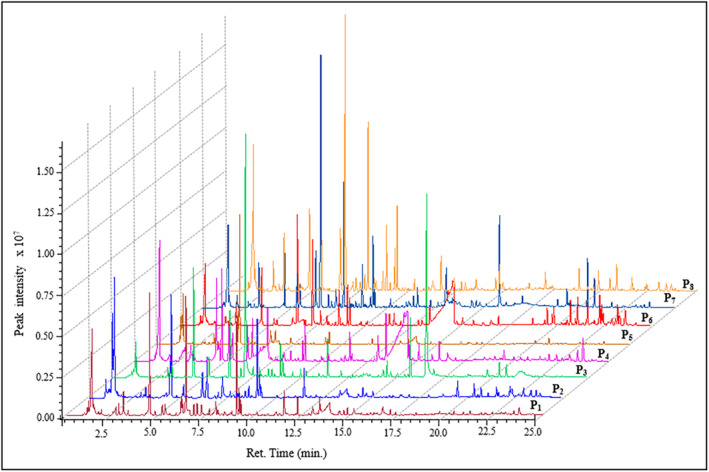
Gas chromatography–mass spectrometry (GC–MS) chromatograms obtained by SPME technique from the tested propolis samples. P_1_, Propolis of Afyonkarahisar Province; P_2_, Propolis of Bitlis Province; P_3_, Propolis of Hatay Province; P_4_, Propolis of Balıkesir Province; P_5_, Propolis of Çorum Province; P_6_, Propolis of Giresun Province; P_7_, Propolis of Antalya Province; and P_8_, Propolis of Adıyaman Province.

The primary peaks in each of the eight propolis samples corresponding to volatile chemicals were identified using GC–MS analysis. The National Institute of Standards and Technology (NIST) library database (Library ID: NIST02.1) was used to generate a library search report based on the peaks assigned by the MS detector and the total ion chromatogram of each peak. Peaks with 80% purity and above that matched the NIST database were classified as specific chemicals. All analyzed extracts showed good separation in terms of their chemical composition. A comparison of the volatile compounds in each propolis sample is shown in Table 2. The amounts of volatile compounds determined by GC–MS were calculated in micrograms per kilogram (μg/kg) according to the internal standard. The GC–MS chromatograms of the propolis samples are shown in Figure 3.

**TABLE 2:** Percentages and quantities of volatile compounds identified in the tested propolis samples.

Sample	P_1_		P_2_		P_3_		P_4_		P_5_		P_6_		P_7_		P_8_	
Compound	μg/kg	%	μg/kg	%	μg/kg	%	μg/kg	%	μg/kg	%	μg/kg	%	μg/kg	%	μg/kg	%
Ketones																
Acetoin	15.4 ± 2.2^b^	0.8	–	–	8.7 ± 0.7^a^	0.4	–	–	–	–	32.5 ± 2.9^c^	0.6	14.7 ± 0.4^b^	0.1	37.3 ± 2.6^c^	0.6
2‐Acetylfuran	51.6 ± 7.6^abc^	2.7	96.7 ± 9.7^bc^	4.5	–	–	107.6 ± 10.7^c^	1.9	32.9 ± 2.4^a^	6.3	35.7 ± 1.9^ab^	0.7	411.8 ± 52.8^e^	3.9	321.6 ± 37.5^d^	4.9
Methylheptenone	19.7 ± 2.3^b^	1.0	14.3 ± 1.0^b^	0.7	19.7 ± 1.2^b^	0.8	–	–	4.3 ± 0.3^a^	0.8	46.9 ± 4.3^d^	0.9	28.4 ± 3.1^c^	0.3	48.3 ± 4.4^d^	0.7
Acetylbenzene	–	–	–	–	12.4 ± 1.1^a^	0.5	30.7 ± 1.9^b^	0.6	–	–	–	–	–	–	–	–
Camphor	–	–	–	–	7.2 ± 1.2^a^	0.3	–	–	–	–	28.8 ± 0.7^b^	0.5	25.0 ± 1.5^b^	0.2	38.8 ± 3.1^c^	0.6
Pinocarvone	39.4 ± 5.2^b^	2.1	19.1 ± 1.5^a^	0.9	8.7 ± 0.9^a^	0.4	195.5 ± 11.2^d^	3.5	8.7 ± 0.7^a^	1.7	131.5 ± 13.6^c^	2.5	226.3 ± 15.4^e^	2.2	57.8 ± 3.8^b^	0.9
Carvone	–	–	–	–	114.3 ± 13.0^a^	4.7	–	–	–	–	28.8 ± 2.5^a^	0.5	718.9 ± 89.6^b^	6.8	–	–
Hydrocarbons																
Hexane	397.8 ± 51.8^b^	20.9	666.9 ± 63.8^c^	30.7	–	–	–	–	142.7 ± 13.6^a^	27.2	–	–	–	–	–	–
Chloroform	32.8 ± 3.6^a^	1.7	20.2 ± 1.1^a^	0.9	–	–	–	–	–	–	–	–	–	–	69.6 ± 7.0^b^	1.1
Methyl cyclopentane	12.9 ± 1.5^b^	0.7	11.1 ± 1.6^b^	0.5	–	–	–	–	2.4 ± 0.2^a^	0.5	–	–	–	–	–	–
Styrene	31.2 ± 4.6^b^	1.6	–	–	47.5 ± 4.1^b^	2.0	134.6 ± 14.3^c^	2.4	4.4 ± 0.3^a^	0.8	28.8 ± 2.2^b^	0.5	184.3 ± 17.0^d^	1.8	–	–
m‐Cymene	274.8 ± 28.4^f^	14.4	232.3 ± 24.3^e^	10.7	85.0 ± 8.8^bc^	3.5	60.9 ± 4.5^b^	1.1	14.7 ± 3.0^a^	2.8	187.9 ± 17.7^d^	3.5	103.5 ± 8.9^c^	1.0	158.2 ± 15.8^d^	2.4
Aldehydes																
Hexanal	8.4 ± 0.8^a^	0.4	4.5 ± 0.6^a^	0.2	38.8 ± 3.7^c^	1.6	–	–	–	–	–	–	–	–	21.9 ± 1.7^b^	0.3
Benzaldehyde	26.2 ± 3.3^a^	1.4	88.9 ± 5.8^ab^	4.1	790.2 ± 87.5^f^	32.5	366.0 ± 30.2^d^	6.5	14.5 ± 2.0^a^	2.8	572.0 ± 70.5^e^	10.7	226.3 ± 14.3^c^	2.2	127.4 ± 11.5^b^	2.0
Octanal	12.6 ± 1.4^b^	0.7	23.9 ± 1.5^c^	1.1	20.3 ± 2.2^bc^	0.8	35.2 ± 2.7^d^	0.6	3.2 ± 0.3^a^	0.6	–	–	35.2 ± 2.6^d^	0.3	89.3 ± 8.9^e^	1.4
Nonanal	50.9 ± 6.8^b^	2.7	89.5 ± 8.9^c^	4.1	68.1 ± 5.6^b^	2.8	135.9 ± 10.0^d^	2.4	11.1 ± 1.4^a^	2.1	65.7 ± 5.1^b^	1.2	138.7 ± 13.3^d^	1.3	135.5 ± 10.2^d^	2.1
α‐Campholenal	40.3 ± 5.0^b^	2.1	–	–	12.7 ± 0.5^a^	0.5	–	–	–	–	–	–	22.7 ± 1.3^a^	0.2	50.5 ± 5.3^b^	0.8
Decanal	7.0 ± 0.8^a^	0.4	26.6 ± 1.1^ab^	1.2	28.4 ± 1.6^ab^	1.2	887.8 ± 94.1^c^	15.8	5.0 ± 0.6^a^	1.0	17.5 ± 1.1^a^	0.3	45.5 ± 3.3^ab^	0.4	101.1 ± 10.4^b^	1.6
Acids																
Boric acid	–	–	62.6 ± 5.9^b^	2.9	–	–	–	–	–	–	40.7 ± 3.0^a^	0.8	–	–	–	–
Acetic acid	36.3 ± 3.0^a^	1.9	–	–	137.2 ± 11.8^a^	5.6	667.9 ± 75.5^b^	11.9	68.7 ± 5.2^a^	13.1	592.7 ± 57.2^b^	11.1	985.2 ± 71.0^c^	9.4	1051.2 ± 103.1^c^	16.2
Butanoic acid	59.3 ± 5.3^b^	3.1	47.4 ± 2.7^ab^	2.2	20.6 ± 2.2^a^	0.9	28.2 ± 2.5^ab^	0.5	23.2 ± 1.1^a^	4.4	142.8 ± 10.4^c^	2.7	284.4 ± 28.0^e^	2.7	219.0 ± 22.0^d^	3.4
Hexanoic acid	10.3 ± 0.9^bc^	0.5	12.7 ± 0.3^bc^	0.6	7.2 ± 0.8^ab^	0.3	54.4 ± 5.2^d^	1.0	1.7 ± 0.0^a^	0.3	–	–	14.7 ± 1.2^c^	0.1	–	–
Hexyl acetate	–	–	–	–	6.6 ± 0.6	0.3	–	–	–	–	–	–	–	–	–	–
Benzoic acid	14.5 ± 2.1^a^	0.7	–	–	17.9 ± 1.7^a^	0.7	1541.6 ± 99.6^c^	27.4	22.7 ± 1.1^a^	4.3	730.5 ± 82.1^b^	13.7	108.0 ± 11.5^a^	1.0	58.6 ± 3.7^a^	0.9
Nonanoic acid	23.9 ± 1.7^ab^	1.2	9.3 ± 0.8^a^	0.4	534.9 ± 47.6^c^	22.0	61.5 ± 3.2^b^	1.1	4.4 ± 0.4^a^	0.8	17.5 ± 0.8^ab^	0.3	–	–	–	–
Diethyl phthalate	36.6 ± 2.6^c^	1.9	15.4 ± 1.2^ab^	0.7	13.0 ± 1.6^ab^	0.5	23.0 ± 1.3^b^	0.4	6.8 ± 0.0^a^	1.3	–	–	154.7 ± 11.0^d^	1.5	23.4 ± 1.0^b^	0.4
Isopentyl acetate	32.3 ± 3.2^a^	1.7	48.2 ± 4.3^a^	2.2	–	–	359.6 ± 28.2^c^	6.4	10.4 ± 0.7^a^	2.0	30.0 ± 2.8^a^	0.6	199.0 ± 20.5^b^	1.9	221.9 ± 21.5^b^	3.4
Alcohols																
1‐Penten‐3‐ol	8.6 ± 0.9^a^	0.5	–	–	–	–	–	–	3.0 ± 0.3^a^	0.6	–	–	77.3 ± 6.1^b^	0.7	–	–
Terpenes																
α‐Thujene	42.2 ± 5.7^b^	2.2	–	–	–	–	–	–	6.0 ± 0.6^a^	1.1	–	–	–	–	–	–
α‐Pinene	318.3 ± 28.8^bc^	16.7	146.8 ± 14.7^ab^	6.8	175.5 ± 13.4^ab^	7.2	110.9 ± 11.3^ab^	2.0	40.1 ± 3.8^a^	7.6	540.7 ± 57.1^c^	10.1	2846.3 ± 257.4^e^	27.0	1301.0 ± 119.7^d^	20.0
Camphene	27.0 ± 1.5^b^	1.4	–	–	–	–	24.3 ± 1.1^b^	0.4	–	–	10.0 ± 1.1^a^	0.2	95.5 ± 9.6^d^	0.9	65.2 ± 6.0^c^	1.0
Verbenene	30.2 ± 3.6^c^	1.6	12.7 ± 0.4^b^	0.6	20.6 ± 2.0^b^	0.8	44.2 ± 2.8^d^	0.8	2.3 ± 0.0^a^	0.4	–	–	46.6 ± 3.8^d^	0.4	65.9 ± 6.1^e^	1.0
β‐Pinene	17.6 ± 1.3^a^	0.9	14.1 ± 1.8^a^	0.6	22.6 ± 1.9^a^	0.9	–	–	–	–	72.6 ± 7.0^a^	1.4	824.7 ± 102.7^b^	7.8	783.1 ± 74.2^b^	12.0
Myrcene	–	–	–	–	–	–	–	–	–	–	–	–	35.2 ± 3.2^a^	0.3	52.7 ± 4.7^a^	0.8
3‐Carene	–	–	34.1 ± 2.8^a^	1.6	20.8 ± 1.5^a^	0.9	–	–	–	–	182.3 ± 14.1^b^	3.4	304.8 ± 35.0^c^	2.9	320.8 ± 34.9^c^	4.9
α‐Terpinene	8.4 ± 0.7	0.4	–	–	–	–	–	–	–	–	–	–	–	–	–	–
Limonene	72.3 ± 7.8^b^	3.8	74.0 ± 7.7^b^	3.4	55.9 ± 3.7^ab^	2.3	104.4 ± 11.^0bc^	1.9	6.8 ± 0.0^a^	1.3	137.8 ± 11.0^c^	2.6	464.1 ± 56.7^e^	4.4	337.7 ± 37.4^d^	5.2
1,8‐Cineole	38.9 ± 2.4^b^	2.0	33.5 ± 2.1^b^	1.5	11.6 ± 0.7^a^	0.5	–	–	27.6 ± 0.6^b^	5.3	–	–	116.0 ± 10.6^c^	1.1	–	–
γ‐Terpinene	–	–	–	–	17.7 ± 1.3^a^	0.7	–	–	–	–	–	–	72.8 ± 6.3^c^	0.7	46.8±3.3^b^	0.7
α‐Pinene oxide	10.5 ± 0.6^a^	0.6	–	–	–	–	–	–	–	–	–	–	75.0 ± 6.1^b^	0.7	–	–
cis‐p‐Menth‐2‐en‐1‐ol	21.6 ± 2.2^a^	1.1	–	–	–	–	146.7 ± 8.2^b^	2.6	8.8 ± 0.0^a^	1.7	48.8 ± 2.9^a^	0.9	594.9 ± 52.9^c^	5.6	–	–
Verbenone	13.6 ± 1.0^a^	0.7	10.9 ± 1.1^a^	0.5	11.0 ± 1.4^a^	0.5	–	–	–	–	–	–	52.3 ± 4.7^b^	0.5	52.7 ± 4.8^b^	0.8
β‐Cyclocitral	15.0 ± 0.6^b^	0.8	19.7 ± 0.9^b^	0.9	–	–	67.9 ± 5.1^d^	1.2	5.0 ± 0.9^a^	1.0	40.7 ± 3.0^c^	0.8	–	–	–	–
Fenchyl acetate, endo‐	–	–	–	–	–	–	89.1 ± 8.6^c^	1.6	5.0 ± 0.4^a^	1.0	–	–	23.8 ± 0.9^b^	0.2	30.0 ± 3.8^b^	0.5
Bornyl acetate	11.9 ± 0.7^a^	0.6	9.3 ± 1.2^a^	0.4	14.2 ± 1.5^a^	0.6	–	–	4.1 ± 0.3^a^	0.8	54.5 ± 3.9^b^	1.0	62.5 ± 6.1^b^	0.6	87.9 ± 7.8^c^	1.4
β‐Bourbonene	–	–	–	–	19.1 ± 1.8^a^	0.8	–	–	–	–	63.9 ± 5.9^b^	1.2	–	–	105.4 ± 8.8^c^	1.6
α‐Ylangene	–	–	–	–	–	–	–	–	–	–	27.5 ± 1.3	0.5	–	–	–	–
α‐Copaene	3.5 ± 0.2^a^	0.2	48.2 ± 4.6^b^	2.2	–	–	50.6 ± 4.1^b^	0.9	–	–	103.3 ± 6.8^c^	1.9	131.9 ± 14.3^d^	1.3	12.4 ± 1.1^a^	0.2
Longifolene	–	–	41.8 ± 4.6^ab^	1.9	28.4 ± 2.4^ab^	1.2	16.0 ± 1.5^a^	0.3	–	–	40.7 ± 2.5^ab^	0.8	389.0 ± 33.1^c^	3.7	54.9 ± 4.2^b^	0.8
Caryophyllene, (E)‐	3.9 ± 0.5^a^	0.2	30.6 ± 2.8^b^	1.4	21.4 ± 2.6^ab^	0.9	–	–	13.4 ± 1.2^ab^	2.6	187.9 ± 12.1^d^	3.5	229.8 ± 20.2^e^	2.2	117.2 ± 8.8^c^	1.8
trans‐a‐Bergamotene	10.0 ± 1.5^a^	0.5	13.5 ± 1.1^a^	0.6	–	–	–	–	–	–	170.4 ± 21.0^b^	3.2	–	–	–	–
α‐Selinene	–	–	6.9 ± 0.6^a^	0.3	–	–	–	–	3.6 ± 0.2^a^	0.7	24.4 ± 2.2^b^	0.5	–	–	–	–
α‐Humulene	–	–	14.1 ± 1.2^a^	0.6	–	–	–	–	–	–	177.9 ± 13.7^c^	3.3	40.9 ± 2.4^b^	0.4	–	–
β‐Chamigrene	–	–	31.9 ± 2.7^ab^	1.5	–	–	–	–	–	–	–	–	–	–	60.0 ± 6.6^b^	0.9
α‐Amorphene	–	–	37.0 ± 3.1^a^	1.7	–	–	32.0 ± 3.5^a^	0.6	–	–	227.4 ± 19.9^b^	4.3	–	–	–	–
α‐Curcumene	–	–	43.7 ± 3.5^b^	2.0	–	–	–	–	4.0 ± 0.4^a^	0.8	128.4 ± 12.6^c^	2.4	–	–	38.0 ± 3.2^b^	0.6
α‐Muurolene	–	–	35.9 ± 1.7^b^	1.7	–	–	63.4 ± 5.6^d^	1.1	2.5 ± 0.1^a^	0.5	45.7 ± 3.2^c^	0.9	–	–	41.0 ± 3.6^bc^	0.6
α‐Patchoulene	–	–	–	–	–	–	–	–	–	–	45.1 ± 3.0	0.8	–	–	–	–
Germacrene D	–	–	–	–	–	–	54.4±4.1^d^	1.0	2.7 ± 0.1^a^	0.5	119.6 ± 8.4^e^	2.2	14.7 ± 0.4^b^	0.1	42.4 ± 3.2^c^	0.7
δ‐Cadinene	16.9 ± 1.9^a^	0.9	23.9 ± 2.3^ab^	1.1	–	–	124.3 ± 11.5^d^	2.2	8.1 ± 1.0^a^	1.5	158.5 ± 13.7^e^	3.0	55.7 ± 4.0^c^	0.5	37.3 ± 3.2^b^	0.6
Caryophyllene oxide	–	–	–	–	13.3 ± 1.2^a^	0.5	–	–	–	–	–	–	58.0 ± 5.6^c^	0.6	27.1 ± 1.3^b^	0.4
Total	1904.6	100.0	2172.2	100.0	2431.5	100.0	5618.6		524.8	100.0	5338.4	100.0	10,539.1		6510.9	100.0

*Note*: Results are shown as the mean ± standard error (*n* = 3). Values with the same superscript in the same row are not significantly different (*p* < .05).

Abbreviations: –, not detected.

**FIGURE 4:**
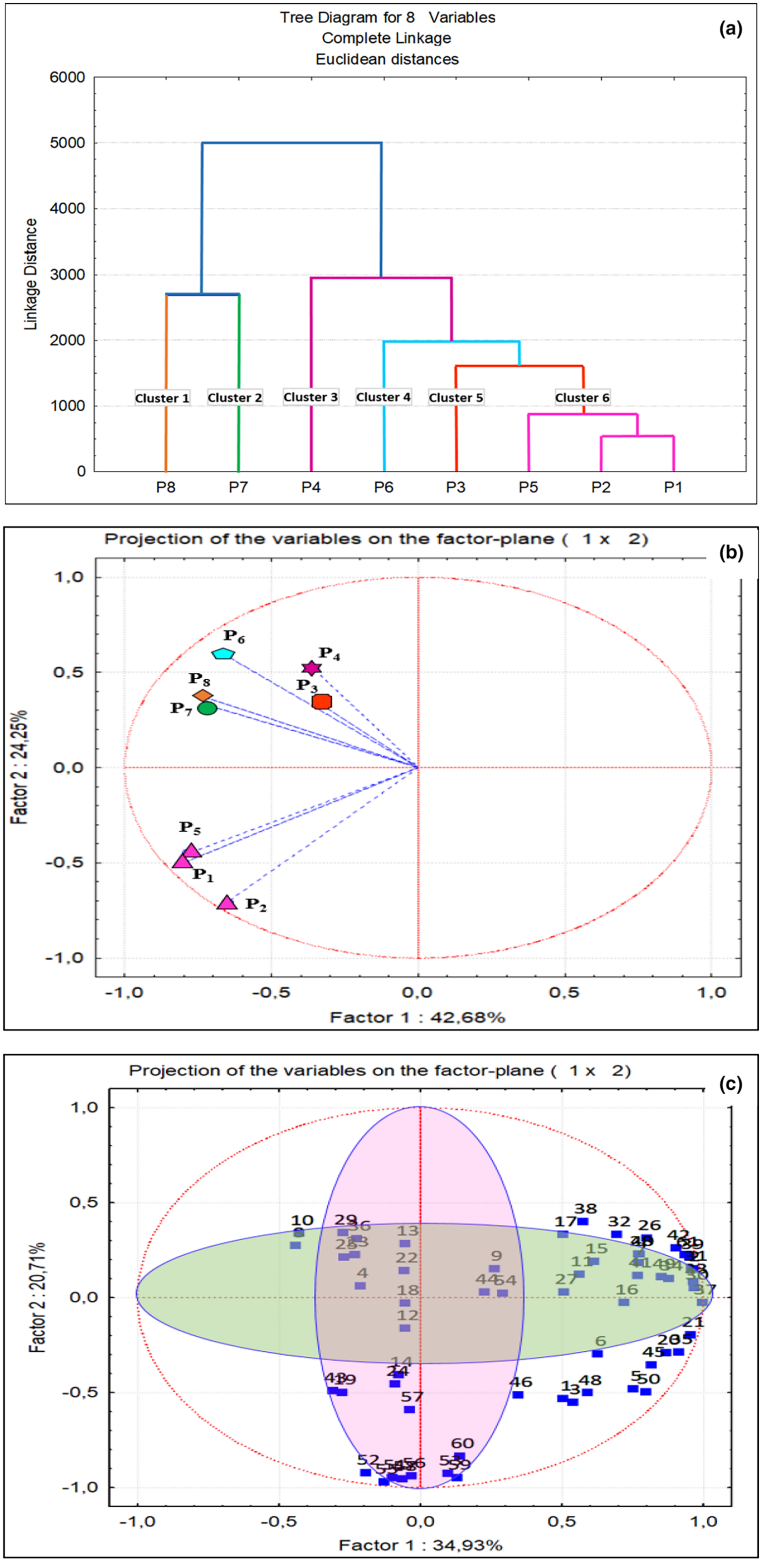
(a) Dendrogram of propolis into clusters drawn using cluster analysis. (b) Projection of cases on the plane spanned by the first and second principal components. (c) Dendrogram for the classification of propolis samples according to their volatile compounds. P1, Propolis of Afyonkarahisar Province, P2, Propolis of Bitlis Province, P3, Propolis of Hatay Province, P4, Propolis of Balıkesir Province, P5, Propolis of Çorum Province, P6, Propolis of Giresun Province, P7, Propolis of Antalya Province, and P8, Propolis of Adıyaman Province. 1, Acetoin; 2, 2‐Acetylfuran; 3, Methylheptenone; 4, Acetylbenzene; 5, Camphor; 6, Pinocarvone; 7, Carvone; 8, Hexane; 9, Chloroform; 10, Metil Siklopentan; 11, Styrene; 12, m‐Cymene; 13, Hexanal; 14, Benzaldehyde; 15, Octanal; 16, Nonanal; 17, α‐Campholenal; 18, Decanal; 19, Boric acid; 20, Acetic acid; 21, Butanoic acid; 22, Hexanoic acid; 23, Hexyl acetate; 24, Benzoic Acid; 25, Nonanoic acid; 26, Diethyl Phthalate; 27, Isopentyl acetate; 28, 1‐Penten‐3‐ol.; 29, α‐Thujene; 30, α‐Pinene; 31, Camphene; 32, Verbenene; 33; β‐Pinene; 34, Myrcene; 35, 3‐Carene; 36, α‐Terpinene; 37, Limonene; 38, 1.8‐Cineole; 39, γ‐Terpinene; 40, α‐Pinene oxide; 41, cis‐p‐menth‐2‐en‐1‐ol; 42, Verbenone; 43, β‐Cyclocitral; 44, Endo‐Fenchyl acetate; 45, Bornyl acetate; 46, β‐Bourbonene; 47, α‐Ylangene; 48, α‐Copaene; 49, Longifolene; 50, Caryophyllene; 51, Trans‐a‐bergamotene; 52, α‐Selinene; 53, α‐Humulene; 54, β‐Chamigrene; 55, α‐Amorphene; 56, α‐Curcumene; 57, α‐Muurolene; 58, α‐Patchoulene; 59, Germacrene D; 60, δ‐Cadinene; 61, Caryophyllene oxide.

**FIGURE 5:**
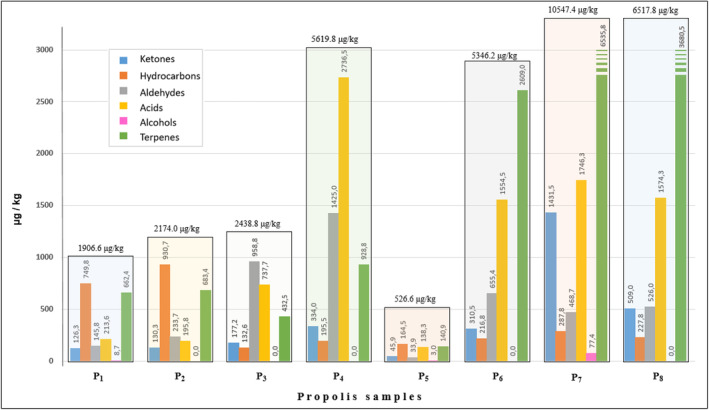
Total volatile amounts of volatile component groups in propolis samples (μg/kg).

A graphical representation of the cluster analysis (CA) of propolis based on its volatile components is shown in Figure 4a. The CA dendrograms of propolis were categorized into six groups, producing results comparable to those of PCA (Figure 4b). The following clusters were identified: Cluster 1 (P8), Cluster 2 (P_7_), Cluster 3 (P_4_), Cluster 4 (P_6_), Cluster 5 (P_3_), and Cluster 6 (P_1_, P_2_, and P_5_). Clusters 1 and 2 displayed the greatest similarity because of the presence of all the compound peaks in the samples. A visual representation of the composition of the states of propolis in the plane, which is composed of the first and second primary elements, is shown in Figure 4c. In all propolis samples, a total of 61 volatile compounds were discovered, comprising 33 terpenes, eight acids, six aldehydes, seven ketones, six hydrocarbons, and one alcohol. Between 29 and 41 volatile compounds were detected in each propolis sample. Sample P_7_ had the highest quantity of volatile chemicals, measuring 10547.4 μg/kg. This was followed by samples P_8_ (6517.8 μg/kg), P_4_ (5619.8 μg/kg), and P_6_ (5346.2 μg/kg). The most prevalent group of compounds was terpenes, with acids being the second most abundant compound group (Figure 5).

Three to six ketone group compounds were detected in the propolis samples at levels of 45.9 and 1431.5 μg/kg (Figure 5). Seven different ketone compounds were identified in varying proportions (between 5.7% and 13.5%) in the propolis samples (Table 2). Acetoin was found to be in the range of 8.7–37.3 μg/kg at P_1_, P_3_, P_6_, P_7_, and P_8_. 2‐Acetylfuran was present at concentrations ranging from 32.9 to 411.8 μg/kg in all samples, except P_3_. Methylheptenone was identified in samples other than P_4_ in amounts ranging from 4.3 to 48.3 μg/kg. Acetylbenzene was detected only in P_3_ and P_4_ at 12.4 and 30.7 μg/kg, respectively. Camphor was present at P_3_, P_6_, P_7_, and P_8_ in amounts ranging from 7.2 to 38.8 μg/kg. Propolis from the Gümüşhane area in Türkiye had 0.4% camphor (Hames‐Kocabas et al., 2013), three propolis samples from five different locations in Greece had 0.2–1.7% camphor (Melliou et al., 2007), and propolis from Ethiopia had 3.23% camphor (Haile et al., 2012). Pinocarvone was detected in all samples, with quantitative results ranging from 0.4% to 3.5% and a corresponding mass range of 8.7–226.3 μg/kg. Previous research has found that the concentration of pinocarvone varies between 0.1% and 4.2% in 17 samples of propolis collected from 39 different locations across Türkiye (Sarıkahya et al., 2021). The percentage of pinocarvone in propolis from Aydın Province of Türkiye was found to be between 0.2% and 0.4% (Sarıkahya et al., 2021), and 0.6% in Gümüşhane Province (Hames‐Kocabas et al., 2013). The carvone content was 4.7%, 0.5%, and 6.8% in P_3_, P_6_, and P_7_, respectively. According to studies conducted by Hames‐Kocabas et al. (2013), carvone was detected in 0.4% of Turkish propolis from the Gümüşhane Province, 0.02–0.47% of Mexican propolis (Pino et al., 2006), and 1.4% of the volatiles in total in Brazilian propolis (Ioshida et al., 2010).

The quantity of volatile compounds belonging to the hydrocarbon group in the propolis samples varied between 132.5 and 930.5 μg/kg, as illustrated in Figure 5. Hexane was present in samples P_1_ (20.9%), P_2_ (30.7%), and P_5_ (27.2%) at 397.8, 666.9, and 142.7 μg/kg, respectively. Chloroform was present to the extent of 32.8 (1.7%), 20.2 (0.9%), and 69.6 g/kg (1.1%) in P_1_, P_2_, and P_8_ samples, respectively. Methyl cyclopentane was detected in P_1_, P_2_, and P_3_ at concentrations of 0.7%, 0.5%, and 0.5%, respectively. The corresponding amounts were 12.9, 11.1, and 2.4 μg/kg. Styrene was present in the range of 4.4–184.3 μg/kg in all samples, except P_2_ and P_8_. Styrene was found to be present in the range of 0.11–4.84% in Uruguayan propolis, 2.3% in Brazilian propolis (Kaškonienė et al., 2014), and 0.04–1.58% in Chinese propolis (Cheng et al., 2013). In our samples, this rate was present in the range of 0.5–2.4%. m‐Cymene was detected in all samples in amounts ranging from 14.7 to 274.8 g/kg. The proportion of the total volatiles varied between 1.0% and 14.4%. Previous research has revealed that the concentration of m‐cymene in Estonian propolis is between 0.14% and 0.21% (Kaškonienė et al., 2014).

Hexanal, benzaldehyde, octanal, nonanal, α‐campholenal, and decanal were identified in propolis samples as aldehyde group volatile compounds. Hexanal was detected in low amounts (4.5–38.8 μg/kg) in P_1_, P_2_, P_3_, and P_8_. In previous research, the concentration of hexanal in Croatian propolis was found to be between 0.2% and 1.9% (Svecnjak et al., 2020), whereas in South African propolis, it reached up to 9.2% (Kamatou et al., 2019). Additionally, Brazilian propolis contains 2.78% hexanal, according to a study by Kaškonienė et al. (2014). Benzaldehyde was found in all samples in varying amounts (14.5–790.2 μg/kg) and ratios (1.4–32.5%). However, in P_3_, the amount was quite high compared to those in the others. Sarıkahya et al. (2021) reported benzaldehyde levels in propolis in various regions of Türkiye, ranging from 0.1% to 10.0% in Aydın, 10.3% in Van, 1.8% in Rize, and 3.5% in Ankara. It was found in the range of 0.2–17.9% in propolis collected from five Croatian islands in the Adriatic Sea (Svecnjak et al., 2020). Cheng et al. (2013) found that benzenaldehyde in 12 Chinese propolis samples ranged from 0.67% to 2.70%. These data are partially consistent with our findings. Octanal was present in the range of 0.3–1.4% in all samples, except P_6_, and varied in quantity from 3.2 to 89.3 μg/kg. Nonanal was found in amounts ranging from 11.1 to 138.7 μg/kg in all samples (*p* < .05). Their ratio to total volatiles ranged from 1.2% to 4.1%. α‐Campholenal ranged from 12.7 to 50.5 μg/kg in P_1_ (2.1%), P_3_ (0.5%), P_7_ (0.2%), and P_8_ (0.8%). Decanal was detected in all samples at amounts ranging from 5.0 to 887.8 μg/kg. Their proportion in the total volatiles ranged from 0.3% to 15.8%. The P_4_ sample had a considerably higher amount than the other samples. In previous studies, it was found to be 0.5% in propolis from Aydın Province and 1.0% in propolis from Van Province of Türkiye (Sarıkahya et al., 2021). Our results are consistent with these findings. In another study, decanal was detected at 0.6% and 6.7% of propolis collected from two different locations in the Gümüşhane Province of Türkiye (Hames‐Kocabas et al., 2013).

Nine different acids were detected in the propolis samples (Table 2). Among the volatile compound groups, acids were mostly found in P_4_ (2736.2 μg/kg), P_7_ (1746.0 μg/kg), P_8_ (1574.1 μg/kg), and P_6_ (1554.2 μg/kg) samples (Figure 5). Boric acid was present in 62.6 g/kg and 40.7 g/kg in P_2_ (2.9%) and P_6_ (2.8%), respectively. Acetic acid was detected in the range of 1.9%–16.1% and in amounts ranging from 36.3 to 1051.2 μg/kg in all samples, except P_2_. Butanoic acid was found in all the samples, ranging from 20.6 to 284.4 μg/kg. Hexanoic acid was present in amounts ranging from 1.7 to 54.4 μg/kg and up to 1.0% in samples other than P_6_ and P_8_. In samples other than P_2_, benzoic acid was found in the range of 0.7%–27.4%. They were found to be between 14.5 and 1541.6 μg/kg in quantity. Nonanoic acid was found in varying amounts (4.4–534.9 μg/kg) and ratios (0.3–22.0%) in all samples (*p* < .05), except P_7_ and P_8_. The acid content was significantly higher in sample P_3_ than in the other samples. Diethyl phthalate was present in varying amounts (6.8–154.7 μg/kg) and at varying levels (0.4–1.9%) in all samples, except P_6_. Previous research on propolis from several locations of Türkiye found the following acid ratios in its volatiles: acetic acid; Aydın 0.1%, Van 0.2% (Sarıkahya et al., 2021), butanoic acid; Aydın 0.1% (Sarıkahya et al., 2021), Rize 0.24–1.09% (Ertürk et al., 2016), benzoic acid; İzmir 0.95%, Balıkesir 1.29%, Bursa 0.97%, Bilecik 1.26%, İstanbul 0.23%, Çanakkale 0.57% (Keskin et al., 2020), Bursa 0.96%, Bartın 1.20%, Ankara 0.53%, Trabzon 4.3% (Uzel et al., 2005), Düzce 2.7% (Donmez, 2020), Rize 0.33% (Ertürk et al., 2016), Ardahan 3.85%, and Erzurum 0.62% (Arslan et al., 2021), and nonanoic acid; Gümüşhane 0.2–0.4%, Erzican 0.4% (Hames‐Kocabas et al., 2013). Such a variation in volatile component profiles is a predicted scenario. Numerous factors, such as bee species, vegetation, time of collection, location, and extraction methods of volatile components, influence the profile of the volatile components of propolis. In samples P_1_, P_5_, and P_7_, 1‐penten‐3‐ol was found to be present as 8.6, 3.0, and 77 μg/kg, respectively.

Several terpene compounds, varying in number from 13 to 21, were detected in these samples. Terpenes are the most prevalent group of volatile compounds, surpassing all the other compound groups. These were highest in samples P_7_ (6535.8 μg/kg), P8 (3680.5 μg/kg), and P_6_ (2609.0 μg/kg) (Figure 5). In general, the proportionally dominant terpenic compounds in the samples included α‐pinene, β‐pinene, limonene, longifolene, and 1,8‐cineole. α‐Pinene was detected in all samples in the range of 40.1–2846.3 μg/kg (proportionally 2.0–27.0%). It was the highest in samples P_7_ (2846.3 μg/kg) and P_8_ (1301.0 μg/kg). The following α‐pinene ratios were discovered in propolis collected from several provinces in Türkiye in previous studies: 2.92% in Bingöl propolis (Arserim‐Ucar et al., 2020), 31.3–55.1% in Aydın propolis, 76.8% in Antalya propolis, 2.3% in Van propolis, 7.5–53.0% in Rize propolis (Sarıkahya et al., 2021), 0.53% in Hatay propolis, and 1.08% in Mersin propolis (Sahinler & Kaftanoglu, 2005). Additionally, propolis samples from five Croatian islands in the Adriatic Sea were found to have a range of 0.6–52.7% (Svecnjak et al., 2020); two propolis samples from five locations in Greece had a range of 7.9% and 45.8% (Melliou et al., 2007); and propolis samples from four different regions of Tunisia had a range of 45.2–90.7% (Jihene et al., 2018). Verbenene was detected in amounts ranging from 2.3 to 65.9 μg/kg and at rates ranging from 0.4 to 1.6% in all samples, except P_6_. The concentration of verbenene in propolis from Hakkari Province, Türkiye, was reported to be between 0.02 and 5.7% by Ecem Bayram (2015). β‐Pinene ranged from 14.1 to 824.7 μg/kg in propolis, except for P_4_ and P_5_. There were no statistically significant differences between the amounts in P_1_, P_2_, P_3_, and P_6_. However, the amounts of β‐pinene in P_7_ and P_8_ were considerably higher than those in the other samples. The β‐pinene in the samples ranged from 0.6% to 12% of all volatile compounds. Sarıkahya et al. (2021) reported that β‐pinene was present in propolis from Aydın Province at 9.4% and 6.7%, in Rize Province at 18.4%, and in İzmir Province at 7.1%, in Türkiye. 3‐Carene was detected in the range of 20.8–320.8 μg/kg (0.9–4.9%) in samples P_2_, P_3_, P_6_, P_7_, and P_8_. Limonene was determined proportionally in the range of 1.3–5.2% and quantitatively in the range of 6.8–464.1 μg/kg. Sarıkahya et al. (2021) detected limonene in the ranges of 2.2–8.8% in propolis from Aydın, up to 21.5% in propolis from Artvin, 2.3–31.5% in propolis from Manisa, and 1.7–19.4% in propolis from İzmir in Türkiye. According to Kaškonienė et al. (2014), limonene was found to be present in 1.13% of Brazilian propolis, 2.43% in Estonian propolis, and 2.08–15.58% in three different varieties of Uruguayan propolis. 1,8‐Cineole (Eucalyptol) was present in P_1_ (2.0%), P_2_ (1.5%), P_3_ (0.5%), P_5_ (5.3%), and P_7_ (1.1%) samples in amounts ranging from 11.6 to 116.0 μg/kg (*p* < .05). 1,8‐Cineole, also known as eucalyptol, was discovered to have a concentration of up to 6.89% in propolis collected from Hakkari, Türkiye (Ecem Bayram, 2015), 25.95% in Estonian propolis (Kaškonienė et al., 2014), up to 6.51% in Chinese propolis (Yang et al., 2010), and up to 11.0% in South African propolis (Kamatou et al., 2019). Bornyl acetate, the acetate ester of borneol used as a food additive, flavoring, and fragrance agent, was present in all samples, except P_4_ in amounts ranging from 4.1 to 87.9 μg/kg. Their proportion of total volatile compounds varied between 0.4 and 1.4%. Of the 39 propolis samples collected from various sites in Türkiye, 17 of them contained bornyl acetate at a ratio of 0.1 to 25.1% (Sarıkahya et al., 2021). Furthermore, the concentration of bornyl acetate in propolis has been reported to be within the range of 0.9–1.8% in Greek propolis (Melliou et al., 2007), 0.1–1.8% in Portuguese propolis (Falcão et al., 2016), and up to 3.2% in South African propolis (Kamatou et al., 2019). α‐Copaene was detected in amounts ranging from 3.5 to 31.9 μg/kg in all samples, except P_3_ and P_5_. Its proportion in all the volatiles ranged from 0.2% to 2.2%. In a previous investigation, α‐copaene was detected in nine out of 39 regions in Türkiye, with concentrations ranging from 0.1% to 1.2% (Sarıkahya et al., 2021). In a separate study, the concentration of α‐copaene in propolis from Bingöl Province (Türkiye) was 4.47% (Arserim‐Ucar et al., 2020). Longifolene (Junipene) was found in the range of 16.0–389.0 μg/kg in all samples, except P_1_ and P_5_. There were no significant differences among the amounts of P_2_, P_3_, and P_6_. The ratio of longifolene to total volatile compounds in the samples ranged from 0.3 to 3.7%. Longifolene ratios in propolis from different cities of Türkiye were previously shown to be 9.1% in Malatya (Yildirim et al., 2004), 0.65% in Mersin (Sahinler & Kaftanoglu, 2005), 0.9–2.75% in Aydın, 1.3% in Manisa, 1.7% in Burdur, and 0.9% in Muğla (Sarıkahya et al., 2021). Caryophyllene was detected in varying amounts (3.9–229.8 μg/kg) and ratios (0.2–3.5%) in all samples, except P_4_. In previous studies, caryophyllene was detected in propolis collected from different cities in Türkiye: Erzurum 0.6% (Arslan et al., 2021), Gümüşhane 0.8%, Erzincan 1.2% (Hames‐Kocabas et al., 2013), and Mersin 1.12% (Sahinler & Kaftanoglu, 2005). α‐Curcumene was detected between 4.0 and 128.4 μg/kg in P_2_ (2.0%), P_5_ (0.8%), P_6_ (2.4%), and P_8_ (0.6%) samples. α‐Muurolene was present in P_2_, P_4_, P_5_, P_6_, and P_8_ prolises in the range of 2.5–63.4 μg/kg. Its proportion in total volatile compounds varied between 0.5 and 1.7%. Germacrene‐D was detected in varying amounts (2.7–119.6 μg/kg) and ratios (0.1–2.2%) in propolis P_4_, P_5_, P_6_, P_7_, and P_8_. δ‐Cadinene was present in amounts ranging from 8.1 to 158.5 μg/kg in all samples, except P_3_. The proportion of total volatiles in the propolis samples ranged from 0.5% to 3.0%. Twelve out of the thirty‐nine propolis samples collected from various places throughout Türkiye had δ‐cadinene levels between 0.1 and 1.7% (Sarıkahya et al., 2021). The percentages were as follows: 5.6% for Erzincan propolis, 0.2% for Gümüşhane propolis (Hames‐Kocabas et al., 2013), and 6.69% for Bingöl propolis (Arserim‐Ucar et al., 2020).

The volatile component ratios differed between the regions, as shown. These findings are partially aligned with our findings. In fact, because the overall amount of volatile components found in each propolis may vary, proportionate data may not yield reliable results. We also performed a quantitative calculation of the data; however, nearly all the research in the literature reported the proportion of volatile components, which complicates comparability. Furthermore, these findings revealed that geographical location, vegetation, and climatic conditions play an important role in the volatile components of propolis. In addition, of course, other factors may also influence the volatile component profile of propolis. These include the bee species, time of propolis collection, collection techniques, and extraction methods.

## 4 CONCLUSIONS

This study assessed the composition of volatile components, antioxidant activity, and total phenolic content of propolis samples collected from eight Turkish districts. The findings showed that the Turkish propolis samples had high concentrations of α‐pinene, β‐pinene, 3‐carene, limonene, 2‐acetylfuran, benzaldehyde, acetic acid, benzoic acid, longifolene, isopentyl acetate, m‐cymene, styrene, and δ‐cadinene. Using principal component analysis and volatile component content as parameters, the samples were well clustered. The propolis samples were sorted according to the provinces in which they were gathered and separated from each other using the first two principal components. Our findings unequivocally demonstrate the remarkable antioxidant properties of Turkish propolis samples. In summary, propolis extracts have the potential to enhance human health in conjunction with pharmaceutical products by supporting the antioxidant defense system in thwarting the production of free radicals. Additionally, they can be employed as oxidation inhibitors or delaying agents to prolong the shelf life of foods. According to our research, the antioxidant activity of propolis is most likely the result of several different chemicals working together, rather than just one.

## AUTHOR CONTRIBUTIONS

**Berfin Yavaş:** Investigation (equal); methodology (equal); validation (supporting); visualization (supporting). **Ayhan Baştürk:** Conceptualization (lead); project administration (lead); writing – review and editing (lead).

## CONFLICT OF INTEREST STATEMENT

The authors declare that they have no known competing financial interests or personal relationships that could have influenced the work reported in this study.

## Data Availability

Data will be made available on request.
